# Does High Thoracic Epidural Analgesia with Levobupivacaine Preserve Myocardium? A Prospective Randomized Study

**DOI:** 10.1155/2015/658678

**Published:** 2015-03-31

**Authors:** Serife Gokbulut Bektas, Sema Turan, Umit Karadeniz, Burcin Ozturk, Soner Yavas, Dilan Biricik, Gul Sevim Saydam, Ozcan Erdemli

**Affiliations:** ^1^Department of Anesthesiology and Reanimation, Turkiye Yuksek Ihtisas Education and Research Hospital, Ankara, Turkey; ^2^Yuksek Ihtisas Education and Research Hospital, kızılay sokak No. 4, Altındag , Sıhhıye, 06810 Ankara, Turkey; ^3^Department of Cardiovascular Surgery, Turkiye Yuksek Ihtisas Education and Research Hospital, Ankara, Turkey; ^4^Department of Biochemistry, Turkiye Yuksek Ihtisas Education and Research Hospital, Ankara, Turkey

## Abstract

*Background*. Our study aimed to compare HTEA and intravenous patient-controlled analgesia (PCA) in patients undergoing coronary bypass graft surgery (CABG), based on haemodynamic parameters and myocardial functions. *Materials and Methods*. The study included 34 patients that were scheduled for elective CABG, who were randomly divided into 2 groups. Anesthesia was induced and maintained with total intravenous anesthesia in both groups while intravenous PCA with morphine was administered in Group 1 and infusion of levobupivacaine was administered from the beginning of the anesthesia in Group 2 by thoracic epidural catheter. Blood samples were obtained presurgically, at 6 and 24 hours after surgery for troponin I, creatinine kinase-MB (CK-MB), total antioxidant capacity, and malondialdehyde. Postoperative pain was evaluated every 4 hours until 24 hours via VAS. *Results*. There were significant differences in troponin I or CK-MB values between the groups at postsurgery 6 h and 24 h. Heart rate and mean arterial pressure in Group 1 were significantly higher than in Group 2 at all measurements. Cardiac index in Group 2 was significantly higher than in Group 1 at all measurements. *Conclusion*. Patients that underwent CABG and received HTEA had better myocardial function and perioperative haemodynamic parameters than those who did not receive HTEA.

## 1. Introduction

The frequency of myocardial ischemia following coronary bypass graft surgery is between 5% and 40%. An increase in the level of response hormones, such as epinephrine and norepinephrine, and cardiac sympathetic activation in the early postoperative period due to the stress of cardiopulmonary bypass are among the more important causes of myocardial ischemia [1–4]. Myocardial and coronary arteries originate from T1–T5, which are innerved by sympathetic nerve fibers [5]. High thoracic epidural analgesia (HTEA) blocks the afferent and efferent fibers of the cardiac sympathetic nerves, increases the diameter of the stenotic coronary epicardial segments in the coronary arterioles, decreases myocardial oxygen consumption, improves left ventricular function, positively affects collateral blood flow during myocardial ischemia, and increases blood flow from the endocardium to the epicardium [4, 6–9]. In this study, we aimed to compare the effects of the HTEA and intravenous patient-controlled analgesia (PCA) on haemodynamic parameters and markers for myocardial ischemia in patients undergoing coronary bypass graft surgery.

## 2. Material and Methods

The study was conducted in accordance with the Declaration of Helsinki and with the approval of the Turkey High Education and Research Hospital Ethics Committee. All patients were informed about the study protocol and provided written informed consent. The study included 34 patients scheduled to undergo elective coronary artery bypass surgery for treatment of coronary artery disease between 15 February 2009 and 10 August 2011. All the patients were in the ASA II-III risk group (ejection fraction >50%), had not previously undergone coronary bypass surgery, and did not have any contraindications for epidural anesthesia. Patients with a contraindication for epidural catheter and abnormal coagulation parameters (APTT > 40 s, INR > 1.25, and fibrinogen concentration < 1 g L^−1^) and those who had renal or hepatic failure and local anesthetic or opioid allergy were excluded from the study. Patients were randomly divided into 2 equal groups: Group 1 received intravenous PCA and Group 2 received HTEA.

### 2.1. Patient Groups

#### 2.1.1. Group 1

Anaesthesia was induced via administration of fentanyl 10–15 *μ*g/kg/min, midazolam 0.1–0.2 mg/kg/min, and rocuronium bromide 0.6–0.9 mg/kg/min and maintained via administration of 50% O_2_ + air and fentanyl, midazolam, and rocuronium bromide 0.1 *μ*g/kg/min. Morphine was administered for postoperative analgesia 24 h intravenous PCA. Continuous induction + bolus mode 5 mg loading dose, 0.3 mg/kg/h basal infusion, and 1 mg bolus dose were arranged as a 15-minute key period.

#### 2.1.2. Group 2

Induction of anaesthesia was the same as in Group 1 and was maintained via administration of 50% O_2_ + air and midazolam 0.1–0.2 mg/kg/min and rocuronium bromide 0.6–0.9 mg/kg/min. A bolus dose of levobupivacaine (0.1 mL/kg/min of 0.25% levobupivacaine) and fentanyl 2 *μ*g/kg/min were administered via an epidural catheter. Dermatome block was evaluated from T1 to L2 with pinprick and temperature sense measurement. Maintenance infusion was provided via administration of 0.25% levobupivacaine 0.1 mL/kg/h and fentanyl 2 *μ*g/mL/h. Postoperative infusion was continued on a 24 h basis with 0.125% levobupivacaine 0.1 mL/kg/h + fentanyl 2 *μ*g/mL/h. In the event of insufficient postoperative analgesia, 0.125% levobupivacaine 4 mL + fentanyl *μ*g/mL/h was planned.

### 2.2. Patient Preparation

During preoperative evaluation, which took place 24 h prior to surgery, information was provided to all patients concerning postoperative pain, epidural, and analgesia with intravenous infusion. Use of a visual analogue scale (VAS) for evaluating pain was explained as follows: 0 = no pain, 1 = slight pain, and 10 = the most severe pain imaginable.

Patients with a normal hemostasis profile were administered HTEA together with EKG, pulse oximetry, and noninvasive arterial blood pressure monitoring under intensive care conditions 1 d prior to surgery. A 20 mg lidocaine injection for local anaesthesia was administered on and below the skin. With the loss of resistance technique at the T2-T3 or T3-T4 level and at the midline the epidural space was entered with an 18 G Tuohy needle (Portex Epidural Minipack, Smiths Medical ASD, Keene, NH, USA). By advancing the epidural catheter with the Tuohy needle the device was inserted 5 cm into the epidural space. After sufficient aspiration and verification that blood and cerebrospinal fluid were absent, 60 mg of lidocaine was administered to the epidural catheter as a test dose. After the inserted catheter was verified, the entrance point was closed and sterilized. After the patients were lying on their backs for 5–10 min, in order to eliminate the possibility of intrathecal placement the necessary neurological and haemodynamic evaluations were carried out.

As premedication on the day of surgery, patients in both groups were given midazolam intravenously. Premedication was administered to patients for 45 min prior to surgery. Patient demographic data were recorded. Prior to induction of anaesthesia, the radial artery catheter, arterial blood pressure, pulse rate and rhythm, pulse oximetry, and peripheral oxygen saturation were monitored. After induction, the central venous catheter, thermodilution catheter, central venous pressure (CVP), mean arterial pressure (MAP), heart rate, cardiac output, mean pulmonary artery pressure (MPAP), pulmonary capillary wedge pressure (PCWP), and cardiac index were measured preoperatively and at 0, 6, and 24 h after surgery.

Troponin I and creatinine kinase-MB (CK-MB) were measured in each patient to determine the presence of myocardial ischemia and a VAS was administered to each patient to measure postoperative pain. Blood samples were obtained preoperatively and 6 and 24 h after surgery for troponin I, CK-MB, total antioxidant capacity (TAC), and malondialdehyde (MDA) measurement. It has been hypothesized that antioxidants inhibit lipid peroxidation and, therefore, may offer protection against the development of cardiovascular disease via preventing formation of early atherosclerotic lesions; this is why TAC and MDA (final product of lipid peroxidation) were measured in each patient.

### 2.3. Statistical Methods

Statistical analysis was performed using SPSS v.13.0 for Windows. The chi-square test and Fisher's exact chi-square test were used for categorical evaluation. For all nonnominal data the Kolmogorov-Smirnov (Lilliefors) normality test was conducted. For comparison of the 2 groups' data that were within the normal range Student's* t*-test as a parametric test was utilized; for data not in the normal range, as nonparametric test, the Mann-Whitney* U* Test was used. Data are presented as mean ± SD. The level of statistical significance was set at *P* < 0.05.

## 3. Results

Among the 34 patients included in the study, 10 were female (29.4%) and 24 were male (70.6%), and the mean age was 55.64 ± 7.86 years (range: 43–73 years). The groups did not differ in terms of demographic data (Table 1). There was not a significant difference in the basal heart rate between the 2 groups, whereas postsurgery values did differ significantly between the 2 groups. The CPB exit and postsurgery heart rate values were compared with the basal values in each group; the heart rate in Group 1 significantly increased, whereas there was not a significant change in Group 2 (Table 2).

MAP basal and CPB exit values did not differ between the 2 groups, but the postsurgery MAP value significantly decreased in Group 2 (Table 2). As compared to the basal cardiac index, at 6 and 24 h after surgery the cardiac index values were significantly higher in Group 1. As compared to the basal cardiac index, at all other time points measured cardiac index was significantly higher in Group 2. There was not a significant difference in CVP, PCWP, or MPAP between or within groups (Table 2). There was no significant difference in the use of inotropic support and urine output between the two groups.

**Figure 1:**
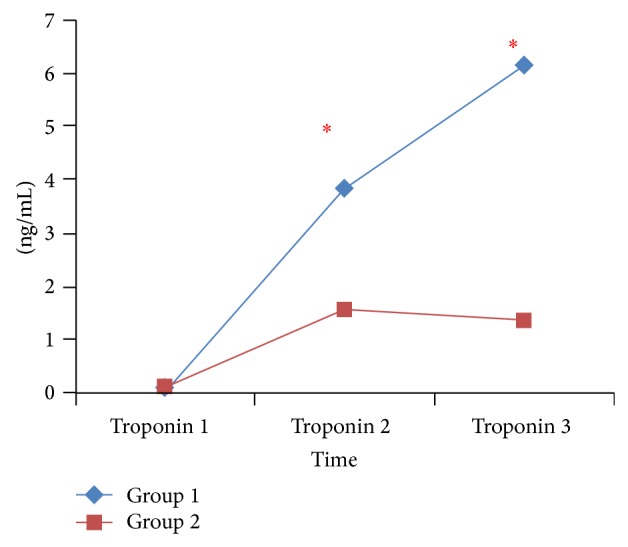
Comparison of troponin values between groups; ^∗^ *P* < 0.05. Group I: general anesthesia, Group II: combined general anesthesia and HTEA with levobupivacaine, troponin 1: preoperatively, troponin 2: 6 hours after arrival in the intensive care unit, and troponin 3: 24 hours after arrival in the intensive care unit.

**Figure 2:**
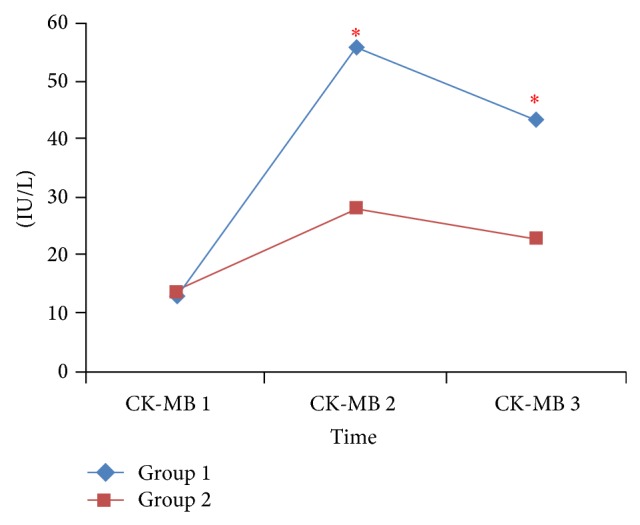
Comparison of CK-MB values between groups; ^∗^ *P* < 0.05. Group I: general anesthesia, Group II: combined general anesthesia and HTEA with levobupivacaine, CK-MB 1: preoperatively, CK-MB 2: 6 hours after arrival in the intensive care unit, and CK-MB3:  24 hours after arrival in the intensive care unit.

**Figure 3:**
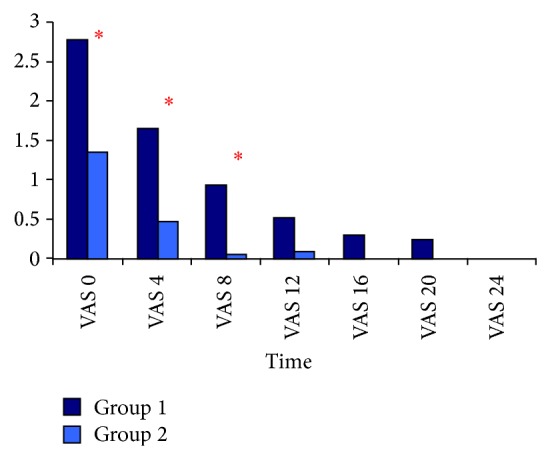
Comparison of VAS values between groups; ^∗^ *P* < 0.05. Group I: general anesthesia, Group II:** c**ombined general anesthesia and HTEA with levobupivacaine, VAS: visual analog scale, VAS 0: arrival in the intensive care unit, VAS 4: 4 hours after arrival in the intensive care unit, VAS 8: 8 hours after arrival in the intensive care unit, VAS 12: 12 hours after arrival in the intensive care unit, VAS 16: 16 hours after arrival in the intensive care unit, VAS 20: 20 hours after arrival in the intensive care unit, and VAS 24: 24 hours after arrival in the intensive care unit.

Although TAC and MDA measurements did not differ significantly between the 2 groups, postoperative TAC was significantly lower and MDA was significantly higher than at baseline in both groups (Table 3). There was not a significant difference in baseline troponin I values between the 2 groups; however, 6 and 24 h after surgery troponin I value was significantly lower in Group 2 (Figure 1). CK-MB baseline values did not differ significantly between the 2 groups; however, at 6 and 24 h after surgery CK-MB was significantly lower in Group 2 (Figure 2). VAS pain scores at 0, 4, and 8 h after surgery were significantly lower in Group 2 but did not differ significantly between groups at other measurement times after surgery (Figure 3).

Additional analgesic was required in 5 patients at 0 h after surgery and in 2 patients at 4 h after surgery in Group 1. Epidural hematoma, nausea, vomiting, and any limitations on walking were not observed in any of the patients.

## 4. Discussion 

Myocardial ischemia following cardiac surgery can occur due to cardiac sympathetic nervous system activation. Sympathetic nervous system activation disrupts the balance between coronary artery blood flow and myocardium oxygen need. This imbalance continues during the early postoperative period and together with unsuitable analgesia increases myocardial ischemia frequency [1, 3, 10–12]. As such, use of a thoracic epidural catheter for administration of postoperative analgesia provides both postoperative analgesia and inhibition of the sympathetic nervous system [13, 14], reducing the risk of myocardial ischemia.

Myocardial and coronary arteries are extensively innervated by sympathetic nerve fibers that originate from T1–T5, which directly provides not only the heart's chronotropic and inotropic control but also total coronary blood flow and distribution [15]. With the thoracic epidural analgesia (TEA) method, the sympathetic activation that originates at mid-T1–T5 is inhibited. Loick et al. [16] evaluated the effect of TEA on haemodynamic parameters and reported that the heart rate in patients decreased significantly during the postoperative period following administration of TEA, as compared to preoperative values, but there was not a difference between the patient and control groups. In the present study HTEA resulted in a lower heart rate and MAP during the postoperative period than those observed in the intravenous PCA group. This result shows the advantage of HTEA over intravenous PCA by means of decreased heart rate and improved coronary blood flow.

Kessler et al. [17] compared the heart rate in patients between those who received general anaesthesia together with TEA (Group 1) and those who received only general anaesthesia (Group 2) during coronary artery bypass surgery performed on a beating heart and reported that the heart rate in the group 1 was lower than preoperative values, during sternotomy and anastomosis compared to group 2. The heart rate during anastomosis was much lower in general anaesthesia together with TEA. In that study intravenous esmolol was administered in the group that received general anaesthesia because of a high heart rate. In that study the intraoperative heart was significantly higher than the basal value in Group 1. In Group 2 there was not a difference between the intraoperative heart rate and the basal rate; however, the heart rate was significantly lower than that in the general anaesthesia group; Kessler et al. study results are consistent with our results.

Licker et al. [18] examined the effect of TEA on cardiovascular autonomy following thoracic surgery and reported that in the TEA treatment group heart rate variables were better restored after surgery. Fillinger et al. [19] and Berendes et al. [20] did not observe a difference in haemodynamic findings between the control group and the TEA treatment group. In the present study as well blood pressure was significantly lower in Group 2 than in Group 1. In Group 2 blood pressure was significantly lower during perfusion exit and at 0 h after surgery than at baseline, which is similar to the findings of Kessler et al. [17] and Royse et al. [21]. Local anesthetic administration also had an important role in lowering TEA's perfusion exit, as compared to baseline and MAP after surgery. In earlier studies on TEA that used ropivacaine and bupivacaine marked hypotension was observed [22, 23]. It was also reported that use of levobupivacaine for TEA was associated with a lower degree of hypotension [23]. In the present study levobupivacaine was used for TEA. It is known that levobupivacaine has less of inotropic effect than bupivacaine and that it lowers the mean stroke volume more significantly and has much less of a depressant effect on atrioventricular conduction and QRS than bupivacaine [22–25]. TEA provides better sympathetic nervous system control than general anaesthesia; however, while this control is being provided the local anesthetic agent to be used for TEA must be carefully selected in order to prevent unwanted effects on haemodynamic parameters. In the present study there was not a difference in the inotropic support used preoperatively, intraoperatively, postoperatively, or the intravenous fluids administered (colloid, crystalloid, blood, and plasma) between the groups, and although the difference in urination was not significant, there was higher urination in the HTEA group, perhaps due to an increase in renal perfusion in those who received HTEA [26].

Berendes et al. [20] did not observe significant differences in cardiac output, PCWP, or CVP between the general anaesthesia group and the HTEA group.

Loick et al. [16] reported that postsurgery CVP was significantly higher in the HTEA group than at baseline and as compared to the control group but did differ at 0, 12, or 24 h after surgery. On the other hand, they observed that the postsurgery cardiac index was lower at baseline in the HTEA group and lower at 0, 12, and 24 h after surgery than in the control group. The increase in MPAP and PCWP was considered high during surgery, but in observations during postsurgery we could not determine a significant difference [16]. In the present study there was not a significant difference in CVP measured preoperatively, at CPB exit, or at 0, 6, and 24 h after surgery. MPAP and PCWP values were significantly different from basal values in the groups and between the groups at 0, 6, and 24 h.

Previous studies have shown that TEA together with general anaesthesia provided better myocardial protection than general anaesthesia alone [16, 27–29]. In the present study there was not a difference between the 2 groups in terms of the preoperative CK-MB or troponin I levels, whereas CK-MB and troponin I levels at 6 and 24 h after surgery were significantly higher in Group 1. We think that the results of this study were similar to those results of papers mentioned above.

In the present study the postoperative TAC level was significantly lower and the postoperative MDA level was significantly higher than at baseline in both groups. Earlier studies on TAC and postoperative myocardial dysfunction reported that more patients with a low preoperative TAC level had postoperative myocardial dysfunction. In the present study postoperative TAC decline and postoperative MDA increase were similar in both groups. As such, the lower CK-MB and troponin I values observed in Group 2 may be indicative of the positive effect of HTEA [30, 31].

Despite the obvious benefits of thoracic epidural anaesthesia and analgesia, it is related to infrequent but potential serious complications such as epidural infections, persistent neurological injury, and especially epidural haematoma which may theoretically increase with anticoagulation in cardiac surgery. A study among members of the Society of Cardiovascular Anesthesiologists revealed that 7% of all anaesthesiologists add epidural technique over conventional management during cardiac surgery. However, Royse showed the safety of high thoracic epidural analgesia in cardiac surgery as well as noncardiac surgery [32]. Similarly Bracco and Hemmerling reported the risk of catheter-related epidural hematoma in cardiac surgery as 1 epidural hematoma for 12000 epidural catheterization processes [33]. Additionally according to Cochrane database, the frequency of neurological complications was lower in patients receiving TEA compared with those receiving GA alone. We placed an epidural catheter in all of the patients the day before the surgery and did not observe any epidural hematoma or neurological complications like voiding dysfunction and walking limitation. This is consistent with the safety profile of epidural interventions demonstrated in the literature. We think that we should not give up on TEA because of the mentioned rare complications.

VAS pain scores in the present study were lower in Group 2 than in Group 1 at all time points measured. Levobupivacaine + fentanyl were started intraoperatively and administered for 48 h after surgery. In Group 1, on the other hand, following a loading dose after surgery, patients received morphine infusion during the first 24 h after surgery. If additional analgesia was required 4 mL of 0.125% levobupivacaine was administered to those in Group 2 and 1 mg of morphine was administered to those in Group 1. The mean VAS score at 0, 4, and 8 h after surgery in Group 2 was significantly lower than that in Group 1; however, at 16, 20, and 24 h after surgery there was not a significant difference in VAS pain score between the 2 groups. The quality of analgesia in Group 2 was better than that in Group 1, as previously reported [17, 20, 21, 29, 34]. In the present study patients in Group 2 did not require additional analgesia, whereas in Group 1 additional analgesia was required in 5 patients at 0 h after surgery and in 2 patients at 4 h after surgery, which indicates that more effective analgesia was provided in Group 2.

Limitation of this study was a relatively small number of patients involved and the lack of postoperative long-term consequences.

In conclusion, patients who underwent coronary artery bypass surgery with HTEA (including levobupivacaine) had better postsurgery myocardial function and perioperative haemodynamic parameters. Multicentered studies including large number of cases are needed to let the thoracic epidural analgesia be preferred in cardiac surgery.

## Figures and Tables

**Table 1:** Distribution of demographic characteristics of groups.

	Group 1 *n* = 17 (%)	Group 2 *n* = 17 (%)	*P*
Age	56 ± 8.4	54 ± 7.4	NS

Gender			
Female	6 (54.5)	4 (23.5)	NS
Male	11 (45.5)	13 (76.5)	

BSA	1.83 ± 0.14	1.89 ± 0.16	NS
Body weight	75 ± 11.3	80 ± 12.0	

EF	57 ± 5.0	54 ± 5.2	NS

HT	4 (23.5)	7 (41.1)	NS
DM	5 (71.4)	2 (11.7)	NS
COPD	1 (5.8)	2 (11.7)	NS
Thyroid dysfunction	2 (11.7)	0	NS
CVD	1 (5.8)	0	NS
Smoking	2 (11.7)	5 (71.4)	NS

Preoperative drug history			
*β*-blockers	3 (17.6)	3 (17.6)	NS
CCBs	0	1 (5.8)	NS
Nitrates	4 (23.5)	3 (17.6)	NS
ACEIs	3 (17.6)	3 (17.6)	NS

Mortality	—	—	—

Complications	—	—	—

BSA: body surface area, EF: ejection fraction, HT: hypertension, DM: diabetes mellitus, COPD: chronic obstructive pulmonary disease, CVD: cerebrovascular disease, CCB: calcium channel blocker, ACEI: angiotensin converting enzyme inhibitor, and NS: not significant.

**Table 2:** The systemic and pulmonary hemodynamic parameters.

	t1	t2	t3	t4	t5
	Mean ± SD	Mean ± SD	Mean ± SD	Mean ± SD	Mean ± SD
Cardiac index (L·min^−1^·m^−2^)					
Group 1	2.13 ± 0.57	2.58 ± 0.72	2.36 ± 0.70	2.75 ± 0.66^+^	2.81 ± 0.62^+^
Group 2	2.22 ± 0.66	3.09 ± 0.64^∗+^	2.92 ± 0.73^∗+^	3.37 ± 0.44^∗+^	3.25 ± 0.14^∗+^
Mean arterial pressure (mmHg)					
Group 1	77.3 ± 11.21	67.8 ± 6.88^+^	74.0 ± 9.20	78.4 ± 10.69	76.5 ± 6.30
Group 2	75.8 ± 13.53	65.9 ± 8.42^+^	67.3 ± 9.11^∗+^	70.1 ± 9.66^*^	70.5 ± 4.95^*^
Central venous pressure (mmHg)					
Group 1	8.88 ± 1.57	9.35 ± 2.31	9.17 ± 2.32	8.78 ± 0.96	8.97 ± 0.78
Group 2	8.47 ± 1.05	8.82 ± 2.03	8.55 ± 1.83	8.56 ± 0.93	8.60 ± 0.93
Heart rate (beats/min)					
Group 1	69.8 ± 12.83	85.4 ± 12.42^+^	85.8 ± 14.71^+^	93.5 ± 18.45^+^	92.3 ± 14.83^+^
Group 2	69.9 ± 10.23	67.7 ± 9.09^*^	67.4 ± 9.15^*^	69.7 ± 9.26^*^	72.9 ± 4.65^*^
Mean pulmonary artery pressure (mmHg)					
Group 1	21.7 ± 5.59	22.0 ± 5.50	21.2 ± 5.69	21.2 ± 6.28	20.4 ± 5.93
Group 2	19.7 ± 4.38	20.4 ± 3.28	20.1 ± 4.18	19.5 ± 3.87	20.1 ± 4.49
Pulmonary capillary wedge pressure (mmHg)					
Group 1	16.2 ± 4.57	16.2 ± 4.46	16.4 ± 4.59	15.7 ± 5.66	15.7 ± 5.46
Group 2	15.5 ± 4.00	15.4 ± 4.18	15.1 ± 3.62	14.5 ± 4.51	15.4 ± 4.21
Systemic vascular resistance (dyne·sec/cm^5^)					
Group 1	520.8 ± 126.3	419.3 ± 101.7	460.1 ± 111.5	354.2 ± 85.9	414.3 ± 100.5
Group 2	486.5 ± 117.9	212.5 ± 51.5^*^	256.8 ± 62.2^*^	230.2 ± 55.8^*^	247.9 ± 60.1^*^
Pulmonary vascular resistance (dyne·sec/cm^5^)					
Group 1	82.4 ± 20.0	59.6 ± 14.4	56.0 ± 13.5	54.5 ± 13.2	74.4 ± 18.0
Group 2	61.7 ± 14.9	31.3 ± 7.6^*^	37.1 ± 9.0^*^	33.1 ± 8.0^*^	25.2 ± 6.1^*^

Group I: general anesthesia;

Group II: combined general anesthesia and HTEA with levobupivacaine;

t1: preoperatively;

t2: after separation from cardiopulmonary bypass;

t3: arrival in the intensive care unit;

t4: 6 hours after arrival in the intensive care unit;

t5: 24 hours after arrival in the intensive care unit;

^∗^ *P* < 0.05: significant difference between Group I and Group II;

^+^ *P* < 0.05: significant difference between the values when compared with t1 value within the groups.

**Table 3:** The malondialdehyde and total antioxidant capacity levels.

Parameters	t1	t2	t3	t4
	Median (range)	Median (range)	Median (range)	Median (range)
Malondialdehyde (*µ*mol/L)				
Group 1	1.53 (0.74–3.51)	1.49 (0.99–3.32)	1.88 (1.14–5.20)	2.33 (1.44–4.85)^+^
Group 2	1.39 (0.74–2.67)	1.58 (0.99–2.97)^∗+^	2.18 (1.24–5.40)^∗+^	2.48 (1.34–5.40)^∗+^
Total antioxidant capacity (mmol Trolox Eq./L)				
Group 1	2.11 (1.84–2.32)	2.02 (1.71–2.14)^+^	1.49 (0.33–1.77)	1.57 (1.23–1.78)
Group 2	2.05 (1.25–2.46)	1.95 (1.59–2.19)^+^	1.47 (1.30–1.83)^∗+^	1.59 (1.12–1.91)^*^

^**∗**^ *P* < 0.05: significant difference between Group I and Group II;

^+^ *P* < 0.05: significant difference between the values when compared with t1 value within the groups.
